# Mouth-rinses for the prevention of chemotherapy induced oral mucositis in children: a systematic review

**Published:** 2015-04-20

**Authors:** A Hashemi, Z Bahrololoumi, Y Khaksar, N Saffarzadeh, H Neamatzade, E Foroughi

**Affiliations:** 1 Department of Pediatrics, Hematology, Oncology Research Center, Shahid Sadoughi University of Medical Sciences and Health Services, Yazd, Iran.; 2 Department of Pediatric Dentistry, Shahid Sadoughi University of Medical Sciences and Health Services, Yazd, Iran.; 3 Department of Medical Genetics, Shahid Sadoughi University of Medical Sciences and Health Services, Yazd, Iran.

**Keywords:** Cancer, Chemotherapy, Children, Mouthwash, Mucositis

## Abstract

**Background:**

The purpose of this review was to evaluate studies in basic oral care interventions to update evidence based practice guidelines for preventing oral mucositis (OM) in cancer patients undergoing chemotherapy.

**Material & Methods:**

Pub Med database and Google Scholar were searched for all papers published between 2000 and December 2014 in English that were conducted using the search terms including ‘‘mocusitis, chemotherapy, mouth-rinses, oral care, oral care protocol, dental care,dental cleaning, oral decontamination, oral hygiene”, and the combined phrases in order to obtain all relevant studies.

**Results:**

The initial search concluded 151 published papers representing both research and clinical work. Review articles, clinical case reports, literature reviews, and other nonresearch articles were excluded from the review. Following this process, 30 papers remained.

**Conclusion:**

Among these, chlorhexidine, normal saline, sodium bicarbonate, iseganan, benzydamine, sucralfate and Granulocyte macrophage colony-stimulating factor have been used in the form of mouth-rinse for prevention of chemotherapy induced mucositis. However, none of these mouthrinses have been shown to be definitely effective in preventing chemotherapy induced oral mucositis.

## Introduction

Cancer is a public health issue all over the world (1, 2). Both hematologic and solid malignancies have several complications, some arising in the oral cavity (3-8). These complications might be a direct consequence of the nature of the malignancy (3, 4), or an adverse effect of the treatment type (i.e. radiotherapy, chemotherapy, hematopoietic stem cell transplantation or a combination of these treatment modalities (5-8).

Oral mucositis is considered to be a common debilitating side effect of chemotherapy with an incidence rate of 40-100%, depending on the type of malignancy, chemotherapy regimen, chemotherapeutic drug type, age of patient, neutrophil count, and level of oral care(6, 7, 9-12). 

Symptoms of chemotherapy–induced mucositis are first seen 3-5 days after initiation of treatment cycle and reach their peak in 7-14 days. The course of this complication normally takes 3 weeks (13). Chemotherapy induced mucositis may cause some complications. Mucositis and its related pain adversely affect nutrition, speaking, function and quality of life of patients under cancer treatment. Mucositis also make patient susceptible to septicemia especially in neutropenic conditions. Chemotherapy-induced mucositis may consequently result in hospitalization of the patient and therefore increasing treatment cost. It may prevent patient from receive optimal treatment because clinician must restrict chemotherapy drug dosage or modify treatment protocol in order to inhibit mucositis progression. Finally, chemotherapy induced mucositis might result in increased morbidity and mortality rate of affected patients (6, 7, 10, 14-17).

To prevent chemotherapy-induced mucositis, different method and therapeutic agents have been used including basic oral care protocol (brushing, flossing, dental visits before and during the treatment and usage of bland mouth-washes) anti-inflammatory agents, antimicrobial agents, cryotherapy, antiseptic agents, antibiotics, vitamins, cytokines, immune regulator, herbal drugs, etc (18-21). In this review, we evaluated studies relevant to mouthwashes containing different category of agents, which have been studied for their possible effect on prevention of chemotherapy-induced mucositis.

## Material and Methods

In this review article, the US National Library of Medicine’s Pub Med database and Google Scholar were searched for all papers published between 2000 and December 2014 that were conducted using the search terms including ‘‘mocusitis, chemotherapy, mouth-rinses, oral care, oral care protocol, dental care, dental cleaning, oral decontamination, oral hygiene, and the combined phrases in order to obtain all relevant studies. We also used a hand search of references of original studies or reviewed articles on this topic to identify additional studies. Articles were in English language. The initial search yielded 151 published papers representing both research and clinical work. Review articles, clinical case reports, literature reviews, and other non research articles were excluded from the review. Following this process, 30 papers remained.

**Figure 1:**
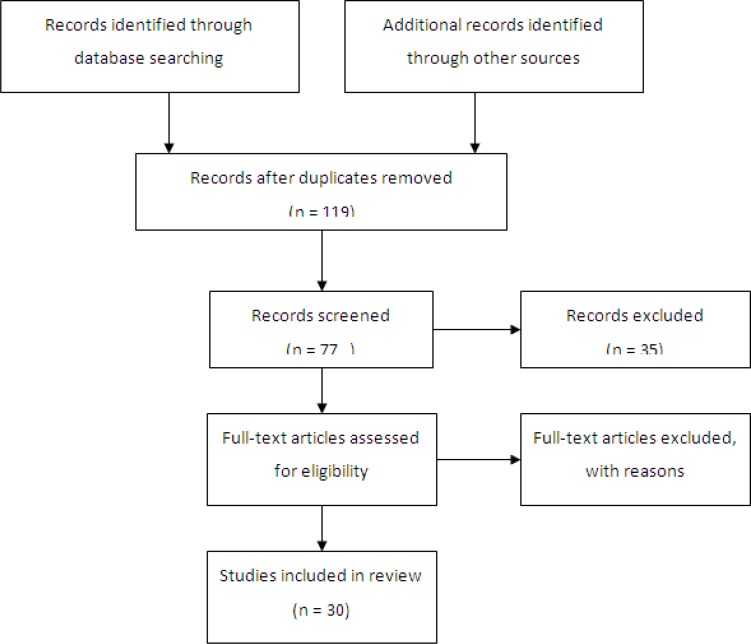
Flow chart of included studies to the review

## Results

The initial search yielded 151 published papers representing both research and clinical work. Review articles, clinical case reports, literature reviews, and other no research articles were excluded from the review. Following this process, 30 papers remained (Figure 1).

## Discussion

We reviewed seven mouthwashes that have been used in chemotherapy induced oral mucositis in children as follow.

**Chlorhexidine**

Chlorhexidine gluconate is a bis-biguanide antimicrobial and antiplaque compound, which has been shown to be both acceptable and well-tolerated in older than 6 year old patients receiving chemotherapy (22). This agent poses high substantivity and is minimally absorbed by gastrointestinal mucous membranes. Chlorhexidine does not have any hazardous adverse systemic effect but if used for a long period, it can lead to reversible discoloration of teeth and mucous membranes (23). Effectiveness of 0.12% and 0.2% chlorhexidine mouth-rinses for prevention of oral mucositis in children and adult population has been widely assessed (24-33).Although most of the articles in this era reported reduction in the incidence of oral mucositis following oral rinsing with chlorhexidine, the results on its effectiveness have not been decisive due to differences in the underlying disease, chemotherapy regimen, studied population, whether or not incorporating the mouth rinse in oral care protocol, concentration of chlorhexidine and frequency of oral rinse. Accordingly, no guideline was able to be published on the utility of chlorhexidine mouthwash for prevention of oral mucositis in both adults and children population receiving chemotherapy. However, it is noteworthy to mention that prescribing chlorhexidine might be beneficial in these patients as it is effective in treatment of gingivitis and plaque control, two common oral diseases in these patients because of their poor oral hygiene (18).

**Benzydamine**

Benzydamine hydrochloride is a non-steroidal anti-inflammatory mouthwash which also poses pain relieving, antimicrobial, antifungal and anesthetic properties (22, 34, 35). Also, it was concluded that in older than 6 year old children, benzydamine is acceptable and well tolerated (22).There is lack of articles on use of benzydamine and as a result no guideline can be published for or against its use in order to prevent chemotherapy induced mucositis. However, in two studies, 0.15% w/v benzydamine hydrochloride has showed to be less effective than 0.2% w/v chlorhexidinegluconate in term of occurrence and severity of oral ulcerations in a pediatric population (26, 36).

**Sodium bicarbonate**

Sodium bicarbonate is a bland mouth rinse that has been shown to be harmless and beneficial for oral hygiene maintenance. H2owever, children might complain from its unpleasant taste (18).There is insufficient published article on the use of sodium bicarbonate mouthwash for preventing oral mucositis in patients under chemotherapy and existing article used sodium bicarbonate in combination with other medications or in patients under both chemo- and radiotherapy (33, 37); therefore it was not possible to draw a guideline in this era. 

**Granulocyte macrophage colony-stimulating factor (GM-CSF)**

GM-CSF is a hematopoietic growth factor which stimulates the development of monocyte/macrophage belonging cells (38).GM-CSF has been shown to promote wound healing in animal studies (39). There are few studies on the effectiveness of GM-CSF mouthrinse in patients under standard or high dose chemotherapy (40-42).In a recent study on the effectiveness of GM-CSF in reducing occurrence of oral mucositis among mixed age group of adolescents and adults, it has not been shown to be beneficial. In this study patients were instructed to rinse for 1 min with 150μg/day of GM-CSF in 100 cm3 of sterile water in four doses per day. Furthermore, patients in both treatment and control groups received conventional prophylaxis with chlorhexidine 0.2% mouthrinse and amphotericin B (40).As insufficient studies are present on the preventive effect of GM-CSF in patients and in the available studies no beneficial effect was observed, it is prescription might not be cost-effective.

**Iseganan**

Iseganan is a structural analog of naturally occurring protegrin-1, a natural peptide isolated from porcine neutrophils. Iseganan also poses microbicidal activity against bacteria and fungi (43-46). Iseganan have been evaluated for its potential effect on reducing chemotherapy induced mucositis in a few studies (47-49).Aphase III prospective, randomized clinical trial on the iseganan did not show any significant efficacy of iseganan in decreasing incidence rate of oral mucositis following stomatotoxic chemotherapy. In the mentioned study, patients were instructed to swish 3ml of 0.3% aqueous solution of iseganan for 2 minutes and then swallow or if not possible expectorate it for 10 days (48). Due to the lack of evidence on efficacy of iseganan oral solutionin patient under chemotherapy, it is not possible to make a recommendation for or against its prescription. 

**Sucralfate**

Sucralfate, a basic aluminum salt of sulfated sucrose, is a cytoprotective and antiulcer agent by its ability to attach to proteins on the surface of ulcers and therefore, form an ulcer adherent complex. This complex in turn, act as a physical barrier and protect mucosal surface of ulcer site by inhibiting its degradation by acid attack (50). Although sucralfate has been incorporated in many studies for prevention of oral mucositis in patients under cancer treatment, most of these studies focused on its preventive effect in patients under treatment with radiotherapy or chemo-radiotherapy (34, 50-64).Some studies have evaluated the efficacy of sucralfate for preventing chemotherapy induced oral mucositis (50, 60-64).As sucralfate has shown limited beneficial effect for the prevention of OM in patients receiving chemotherapy, its prescription is not suggested in these patients(19).

**Normal saline**

Normal saline (sodium chloride 0.9% solution) is a harmless bland isotonic oral rinse which has been shown to be beneficial in maintaining appropriate oral hygiene due to its safety, lowest toxicity and physiologic properties (18). Although there are several studies on the preventive effect of normal saline on oral mucositis in chemotherapy, radiotherapy and/or HSCT patients (32, 65-71), few studies have assessed its effect on prevention of mucositis resulting from chemotherapy (32, 71). Actually, in one of the mentioned studies normal saline showed inferior effect on preventing chemotherapy induced mucositis compared to chlorhexidine and cryotherapy(32). in the other one, normal saline was less effective in preventing chemotherapy induced mucositis in comparison to honey plus normal saline and placebo groups (71).As there is insufficient data in this content and also as normal saline is mostly included in oral hygiene regimens and is mostly not prescribed as single mouth-rinse, the result cannot be decisive for or against use of this mouth rinse in these patients.

## Conclusion

Oral mucositis is a common debilitating adverse effect of chemotherapy in cancer patients. Therefore, it is essential to investigate medications for prevention of this complication. Several researches have been done focused on the effectiveness of mouthrinses containing bland rinses, cytokines, antibacterial and anti-inflammatory agents, etc., for prevention of chemotherapy induced oral mucositis. However, further investigations are required in order to be able to publish a practical guideline in this contex. 
